# Patient Experience Captured by Quality-of-Life Measurement in Oncology Clinical Trials

**DOI:** 10.1001/jamanetworkopen.2020.0363

**Published:** 2020-03-04

**Authors:** Alyson Haslam, Diana Herrera-Perez, Jennifer Gill, Vinay Prasad

**Affiliations:** 1 Knight Cancer Institute, Oregon Health & Science University, Portland; 2 Division of Hematology Oncology, Knight Cancer Institute, Oregon Health & Science University, Portland; 3 Department of Public Health and Preventive Medicine, Oregon Health & Science University, Portland; 4 Center for Health Care Ethics, Oregon Health & Science University, Portland; 5 Division of General Medicine, Department of Medicine, Oregon Health & Science University, Portland

## Abstract

**Importance:**

Quality of life (QoL) is an important consideration in cancer medicine, especially because drugs are becoming more costly and may only result in modest gains in overall survival. However, there has been no descriptive analysis for the points at which QoL is measured in cancer trials.

**Objective:**

To estimate the prevalence of studies that measure QoL at different points and see how many studies measure QoL for the entirety of a patient’s life.

**Design, Setting, and Participants:**

This cross-sectional analysis includes all articles on oncology clinical trials in the 3 highest-impact oncology journals, published between July 2015 and June 2018, that reported QoL outcomes.

**Main Outcomes and Measures:**

Data were abstracted on when QoL was assessed and the characteristics of these studies.

**Results:**

For all 149 studies that met inclusion criteria, QoL assessment was high during treatment (104 articles [69.8%]), during follow-up (81 articles [54.4%]), and after the end of the intervention (68 articles [45.6%]). In 5 of the 149 studies (3.4%), QoL was assessed until death, including in only 1 of the 74 studies on metastatic or incurable cancers. Among these 5 studies, only 1 (20%) used a drug intervention, 1 (20%) used a behavioral intervention, and 2 (40%) used a radiation intervention; only 1 of 5 was in the metastatic setting. The number of studies that reported a positive QoL outcome (ie, QoL outcome was more favorable in the intervention group than in the control group) was between 42 of 81 articles (51.9%) and 16 of 28 articles (57.1%) for most QoL assessment points but only 1 of 5 articles (20%) for studies measuring QoL until death.

**Conclusions and Relevance:**

This study found that most clinical trials assessed QoL during the treatment or intervention and often during a given amount of follow-up but infrequently assessed QoL on disease progression and rarely followed QoL until the end of the patient’s life. Most studies reporting QoL until the end of life reported worse QoL outcomes for the intervention group than the control group. Future research and policy recommendations should consider not just short-term QoL outcomes but QoL outcomes throughout the patient’s cancer care.

## Key Points

**Question:**

How often do oncology studies assess quality of life (QoL) throughout a patient’s disease course?

**Findings:**

This cross-sectional analysis of 149 oncology studies published in high-impact medical and oncology journals found that most studies (69.8%) assessed QoL during the intervention, whereas only 3.4% of studies assessed QoL until the time of death.

**Meaning:**

These findings suggest that many oncology studies only assess QoL during the intervention; future research should consider the long-term outcomes throughout the patient’s life.

## Teaser

This cross-sectional study of published journal articles examines the prevalence of studies that measure patient quality of life at all, and how many of these persist with measurements for the entirety of a patient’s life.

## Introduction

Health-related quality of life (QoL) and other patient-reported outcomes are vital to assessing patient perspective and experience. They reflect patient satisfaction and perceived benefits of an intervention that are not necessarily captured by other end points. These outcomes are commonly used in clinical trials, and regulatory and reimbursement agencies have begun to require these data as part of their evaluation process.^1^

Such QoL outcomes can be especially important in cancer clinical trials, where the intervention may not be designed to cure the disease but may only modestly prolong life. An analysis of 71 consecutively approved cancer drugs for solid tumors found that survival was increased by a median of 2.1 months.^2^ In such cases, improvement in QoL is an important consideration.

One overlooked consideration in the measurement of QoL is that even though drugs are often evaluated for their effects on overall survival across the remainder of a patient’s life, QoL may not be; QoL may only be measured during or at completion of therapy and may not be measured beyond therapy. In other words, the time span over which QoL is measured until the end of life is unknown. This is important because a drug may improve QoL in the short term, but those gains may be offset by worse QoL after therapy is complete, perhaps because of few remaining effective therapies or rapid progression of disease.

For this reason, we sought to characterize QoL measurement in randomized clinical trials (RCTs) in high-impact oncology journals. Specifically, we sought to estimate the prevalence of QoL being measured until the end of life, in addition to the duration of the study intervention or after a short follow-up.

## Methods

### Study Design and Search Strategy

This was a retrospective cross-sectional study that sought all RCTs that reported on QoL, including health-related QoL, in 3 high-impact oncology journals. We adhered to Strengthening the Reporting of Observational studies in EpidemiologyStrengthening the Reporting of Observational Studies in Epidemiology (STROBE) reporting guideline. We selected articles for this analysis from the 3 highest-impact oncology journals, as per impact-factor scores on Scimago Journal and Country Rank, using the most recent years (July 2015 through June 2018) of *Lancet Oncology*, *Journal of Clinical Oncology*, and *JAMA Oncology*. For each of the journals, we searched for the term *quality of life* on the journal’s website, and we limited the search to research articles only. Selected articles needed to (1) be an RCT, (2) have performed the analysis in the originally randomized groups, (3) have evaluated QoL in the study, and (4) have reported the results of the QoL analysis in the study. We excluded research letters, because they did not provide adequate detail on methods, and we excluded studies that combined multiple RCTs. The search was performed on July 2, 2018. Because we used publicly available data, and this is not human subjects research in accordance with 45 CFR §46.102(f), we did not submit this study to an institutional review board or require informed consent procedures.

### Statistical Abstraction

Information abstracted for each article included date of publication; cancer type; setting; whether the cancer under investigation was metastatic, advanced, and/or incurable (yes, no, or not applicable, for studies where the cancer was metastatic but the intervention was designed to test palliative care or not designed to improve duration of life); intervention type (a drug, behavioral intervention, radiation regimen, surgery, treatment algorithm, device, or procedure); whether overall survival was a primary or secondary end point or not indicated; the timing of the QoL assessment; the QoL metric or metrics; whether the QoL assessment was done during the intervention; and the results of the QoL outcome (positive, negative, or indeterminate). We also abstracted the median time to deterioration in QoL and median overall survival for studies that included participants with metastatic, advanced, or incurable cancer. In some cases, we searched for companion studies, when survival metrics were reported in a separate article, or on ClinicalTrials.gov, using the study identifier in the article. Two of a group of 3 reviewers (A.H., D.H-P., and/or J.G.) independently reviewed and abstracted data from each article. A third reviewer from this group adjudicated any discrepancies.

Based on the intervention duration and the timing of the QoL assessments, we abstracted data for 5 different QoL assessment points: during the intervention, at the end of treatment, after some follow-up time after completion of the intervention, until progression of cancer, and until death. In determining whether QoL was assessed at each point, we looked at the timing of reported QoL outcomes and not at the reportedly collected QoL data. Result outcomes were considered positive when the QoL results demonstrated a beneficial outcome or if there was no decline in QoL in the presence of improved disease progression or survival (primary outcome). Results were indeterminate when there were both improvements and declines in different QoL measures. Assessment until progression was affirmative if QoL was measured at the progression of disease or the discontinuation of treatment because of progression. Assessment of QoL until death was recorded as affirmative if either the study specifically stated that QoL was measured until death or until overall survival of the study cohort was less than 50%. Because of the small number of studies reporting on some of the intervention types, some categories were collapsed (eg, treatment algorithms, devices, and procedures were combined into a category called *other* and surgery, radiation, and chemoradiation were combined into a chemotherapy combination category).

Because we were specifically interested in determining whether QoL was reported until death, we wanted to compare median observation time with median overall survival. As a metric for median observation time, we used median time to deterioration. We then calculated median times to deterioration by QoL outcomes. For studies that did not report median time to deterioration and stopped reporting QoL data after progression or recurrence, we used median progression-free survival or median recurrence-free survival as a surrogate for median observation time. For studies that reported QoL on all participants and had set points (eg, 6 and 18 months) for assessing QoL instead of a set frequency, we used the latest period for which there were QoL results reported. For studies reporting QoL by weeks, we converted this value to months by dividing by 4, and when days were reported, we divided by 30, so all values would have the same unit.

### Statistical Analysis

Frequencies were calculated for categorical variables throughout. A χ^2^ test of independence was used to assess differences in study qualities between those that included metastatic or incurable cancers and those that did not. We also used χ^2^ tests to determine global differences in whether or not QoL was assessed (during treatment, at the end of treatment, after follow-up, until disease progression, or until death) for different intervention types and QoL outcomes. The Fisher exact test was used for comparisons where there were fewer than 5 counted items in a category. These methods were also used to determine differences, if any, in the proportion of positive outcomes between the different QoL-assessment periods (all studies and metastatic or incurable cancers only). The statistical analyses were done using R version 3.5.0 (R Project for Statistical Computing) and a 2-tailed *P* value less than .05 as the level of significance.

## Results

There were 856 articles reviewed for inclusion, of which 149 met inclusion criteria.^3,4,5,6,7,8,9,10,11,12,13,14,15,16,17,18,19,20,21,22,23,24,25,26,27,28,29,30,31,32,33,34,35,36,37,38,39,40,41,42,43,44,45,46,47,48,49,50,51,52,53,54,55,56,57,58,59,60,61,62,63,64,65,66,67,68,69,70,71,72,73,74,75,76,77,78,79,80,81,82,83,84,85,86,87,88,89,90,91,92,93,94,95,96,97,98,99,100,101,102,103,104,105,106,107,108,109,110,111,112,113,114,115,116,117,118,119,120,121,122,123,124,125,126,127,128,129,130,131,132,133,134,135,136,137,138,139,140,141,142,143,144,145,146,147,148,149,150,151^ Studies that were excluded were not RCTs or did not analyze data in randomized groups (544 articles), did not report or assess QoL (123 articles), reported QoL in a separate manuscript (38 articles), was a research letter (1 article), or was a study that combined 3 RCTs (1 article). Seventy-four studies included people with metastatic, advanced, and/or incurable cancers (49.7%); 42 studies included patients with cancers that were not metastatic, advanced, or incurable (28.2%); and 33 studies included interventions that were not designed to improve survival (22.1%). (All references are in the eAppendix in the Supplement.)

Among eligible studies of metastatic, advanced, or incurable cancers (Table 1), 40 studies were published in *Lancet Oncology*, 31 studies in the *Journal of Clinical Oncology*, and 3 studies in *JAMA Oncology*. Quality of life was the primary study outcome in 2 studies (4.1%), whereas most studies did not have QoL as a primary end point (72 articles [95.9%]). Most studies used a drug intervention (68 articles [90.7%]). Forty-four studies (60.0%) reported a positive QoL outcome, 24 studies (32.0%) had negative outcomes, and 6 studies (8.0%) had indeterminate findings (eAppendix in the Supplement).

**Table 1.** Characteristics of 149 Studies That Included Quality of Life in 3 High-Impact Medical Journals, July 2015 Through June 2018

Characteristic	Studies, No. (%)	
	On Metastatic, Advanced, or Incurable Cancer	On Nonmetastatic Cancer (or Not Applicable)
Total articles, No.	74	75
Journal^a^		
* Lancet Oncology*	40 (54.0)	25 (33.3)
* Journal of Clinical Oncology*	31 (41.9)	42 (56.0)
* JAMA Oncology*	3 (4.0)	8 (10.7)
Years of publication		
2015	13 (17.6)	16 (21.3)
2016	20 (27.0)	24 (32.0)
2017	26 (35.1)	22 (29.3)
2018	15 (20.3)	13 (17.3)
Quality-of-life assessments		
During intervention^b^		
Yes	66 (89.2)	38 (50.7)
No	8 (10.8)	37 (49.3)
At the end of intervention		
Yes	33 (44.6)	35 (46.7)
No	41 (55.4)	40 (53.3)
After end of intervention, during follow-up^c^		
Yes	32 (43.2)	49 (65.3)
No	42 (56.8)	26 (34.7)
At progression^d^		
Yes	22 (29.7)	6 (8.0)
No	52 (70.3)	68 (90.7)
Not indicated	0	1 (1.3)
Until death^e^		
Yes	1 (1.4)	4 (5.3)
No	71 (95.9)	33 (44.0)
Not indicated	2 (2.7)	38 (50.7)
Quality of life as primary end point		
Yes	2 (2.7)	9 (12.0)
No	72 (97.3)	66 (88.0)
Results^f^		
Positive	44 (59.4)	40 (53.3)
Negative	24 (32.4)	31 (41.3)
Indeterminate		4 (5.3)
Intervention type^b^		
Drug	68 (91.9)	33 (44.0)
Behavior	0	21 (28.0)
Chemotherapy combination	1 (1.3)	8 (10.7)
Radiation	3 (4.1)	9 (12.0)
Surgery	1 (1.3)	1 (1.3)
Other	1 (1.3)	3 (4.0)
Overall survival outcome^c^		
Primary	29 (39.2)	8 (10.7)
Secondary	39 (52.7)	26 (34.7)
Not a main outcome	2 (2.7)	2 (2.7)
Not indicated	4 (5.4)	39 (52.0)

^a^ *P* = .03.

^b^ *P* < .001.

^c^ *P* = .007.

^d^ *P* = .003.

^e^ Numbers for not indicated was too great to derive meaningful comparisons.

^f^ A positive result indicates that patient’s quality of life was better in the intervention group.

Among eligible studies with cancers that were not advanced, metastatic, or incurable and studies that used an intervention not designed to improve survival (Table 1), 25 were published in *Lancet Oncology*,42 in the *Journal of Clinical Oncology*, and 8 in *JAMA Oncology*. Quality of life was the primary study outcome in 10.8% (9 studies), whereas most studies did not have QoL as a primary end point (66 articles [89.2%]). Most studies used a drug intervention (33 articles [44.6%]), 21 studies used a behavioral intervention (27.0%), 9 studies used therapeutic radiation as an intervention (12.2%), 1 study concerned a surgery intervention (1.4%), 8 studies used a chemotherapy regimen (with or without surgery, radiation, or another drug [10.8%]), and 3 studies had some other type of intervention (a device, treatment algorithm, or procedure [4.1%]). The most common QoL outcome was positive (40 articles [52.7%]); 31 studies (41.9%) had negative outcomes, and 4 (5.4%) had indeterminate outcomes (eAppendix in the Supplement).

For all studies and interventions, QoL assessment was high during the intervention (66 articles [89.2%] on metastatic cancers; 38 articles [50.7%] on nonmetastatic cancers), after the end of the intervention (33 articles [44.6%] on metastatic cancers; 35 articles [46.7%] on nonmetastatic cancers), and during follow-up (32 articles [43.2%] on metastatic cancers; 49 articles [65.3%] on nonmetastatic cancers) (Table 1). The assessment of QoL until the time of death was low for studies of both metastatic cancers (1 article [1.4%]) and nonmetastatic cancers (4 articles [5.3%]) (eAppendix in the Supplement).

For studies that measured QoL during treatment, 87 studies (83.7%) used a drug intervention and 8 studies (7.7%) used a behavioral intervention (Table 2). For studies that measured QoL until the end of treatment, 50 studies (73.5%) used a drug intervention and 11 studies (16.2%) used a behavioral intervention. For studies that measured QoL after some amount of follow-up time, 46 studies (56%) used a drug intervention and 14 studies (17.3%) used a behavioral intervention. For studies measuring QoL on progression, 25 studies (89.3%) used a drug intervention and none used a behavioral intervention. For studies that measured QoL until death, only 1 study (20%) used a drug intervention, 1 study (20%) used a behavioral intervention, and 2 studies (40%) used a radiation intervention (eAppendix in the Supplement).

**Table 2.** Frequencies of Intervention Types for Each of the Quality-of-Life Measurements in All Included Randomized Clinical Studies (N = 149) from *Lancet Oncology*, *Journal of Clinical Oncology*, and *JAMA Oncology* from July 2015 Through June 2018^a^

Treatment	Frequency of Assessment of Quality of Life, No. (%)				
	During Treatment (n = 104)^b^	End of Treatment (n = 68)	After Follow-up (n = 81)^c^	Progression (n = 28)	Death (n = 5)^d^
Drug	87 (83.7)	50 (73.5)	46 (56.8)	25 (89.3)	1 (20.0)
Behavior	8 (7.7)	11 (16.2)	14 (17.3)	0 (0)	1 (20.0)
Radiation	2 (1.9)	3 (4.4)	10 (12.3)	2 (7.1)	2 (40.0)
Surgery	0	0	2 (2.5)	0	0
Chemotherapy combination with surgery or a drug	5 (4.8)	3 (4.4)	7 (8.6)	0	1 (20.0)
Other (procedure, device, or treatment algorithm)	2 (1.9)	1 (1.5)	2 (2.5)	1 (3.6)	0

^a^ Comparing global differences in whether or not quality of life was assessed for each point (eg, during treatment, end of treatment) by intervention type.

^b^ *P* < .001 with Fisher exact test.

^c^ *P* = .04.

^d^ Numbers were too few for statistical comparison.

**Figure 1.**
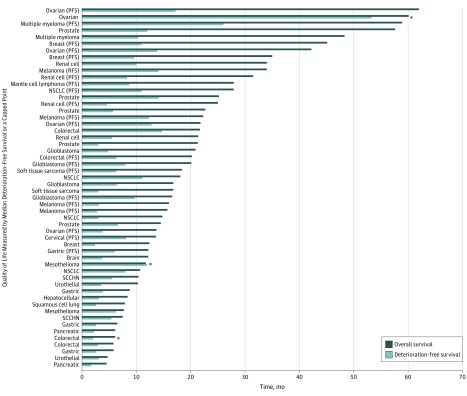
Median Overall Survival and Median or Capped Time of Quality-of-Life Assessment in the Intervention Arm of Studies That Report Quality-of-Life Measures and Include Patients With Metastatic, Advanced, or Incurable Cancers The quality-of-life assessment was capped at a set time in the items marked with an asterisk. NSCLC indicates non–small cell lung cancer; PFS, progression-free survival; RFS, relapse-free survival; SCCHN, small-cell carcinoma of the head and neck.

**Figure 2.**
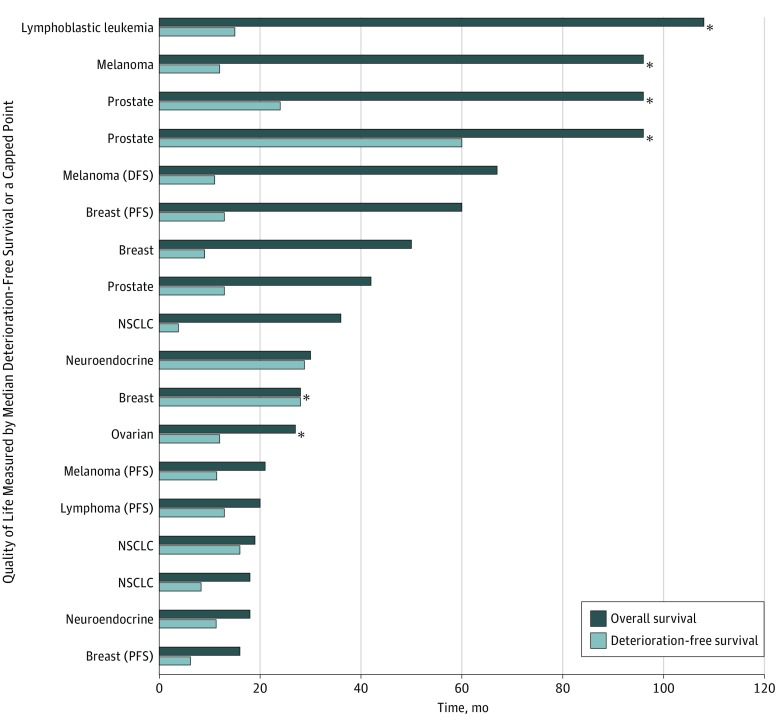
Known Overall Survival and Median or Capped Time of Quality-of-Life Assessment in the Intervention Arm of Studies Reporting Quality-of-Life Measures in Which Patients With Metastatic, Advanced, or Incurable Cancers Were Included and Median Overall Survival Was Not Reached The quality-of-life assessment was capped at a set time for the items marked with an asterisk. NSCLC indicates non–small cell lung cancer; PFS, progression-free survival; RFS, relapse-free survival.

The number of studies that reported a positive QoL outcome was 59 (56.7%) for studies that measured QoL during treatment (Table 3), 35 (51.5%) for studies that measured QoL at the end of treatment, 42 (51.9%) for studies measuring QoL after some amount of follow-up time, 16 (57.1%) for studies measuring QoL on progression, and 1 (20%) for studies measuring QoL until death (eAppendix in the Supplement). Similar patterns in the distribution of positive QoL outcomes were seen for studies that included metastatic, advanced, or incurable cancers. Figure 1 (for studies in which the median overall survival was reached) and Figure 2 (for studies in which the median overall survival was not reached) show the comparison of overall survival and the duration that QoL was assessed in studies that included patients with metastatic, advanced, or incurable cancers.

**Table 3.** Frequencies (Percentages) of Quality-of-Life Outcomes in All Included Randomized Clinical Trials for Each of the Measurement Period in *Lancet Oncology*, *Journal of Clinical Oncology*, and *JAMA Oncology* from July 2015 Through June 2018

Result	Frequency of Assessment of Quality of Life, No. (%)				
	During Treatment	End of Treatment	After Follow-up	Progression	Death
**All Trials (N = 149)**					
No.	104	68	81	28	5
Positive^a^	59 (56.7)	35 (51.5)	42 (51.9)	16 (57.1)	1 (20.0)
Negative	39 (37.5)	26 (38.2)	33 (40.7)	10 (35.7)	4 (80.0)
Indeterminate	6 (5.8)	7 (10.3)	6 (7.4)	2 (7.1)	0
**Trials With Metastatic, Advanced, or Incurable Cancers (n = 74)**					
No.	66	33	32	22	1
Positive^a^	39 (59.1)	16 (48.5)	19 (59.4)	13 (59.10)	0
Negative	22 (33.3)	14 (42.4)	10 (31.2)	7 (31.8)	1 (100)
Indeterminate	5 (7.8)	3 (9.1)	3 (9.4)	2 (9.1)	0

^a^ A positive result indicates that patient’s quality of life was better in the intervention group.

## Discussion

In a systematic sampling of QoL studies in high-impact oncology journals, we found that most studies assessed QoL during the treatment or intervention and often during a given amount of follow-up but often did not assess QoL on progression and rarely assessed QoL until the end of the patient’s life. Specifically, we found that QoL was only measured until the end of life in 1 of the 74 studies assessing QoL among patients with metastatic or incurable cancers. An evaluation of QoL beyond treatment may be especially informative for patients with advanced cancers, because available treatments may offer only marginal survival gains at the expense of potential toxicity or harm.^152^

Assessing QoL until death is particularly noteworthy, considering only 20% of studies that reported QoL until death also reported improvements in QoL with the treatment. In other words, most studies that assessed QoL until the end of life found no QoL benefit from the intervention. Whereas those that measured QoL during treatment reported QoL improvement from the intervention in 56.7% of studies. Those that reported QoL at other points had a similar percentage of positive findings as those that reported QoL during treatment. These results suggest that the typical length of QoL assessment may be inadequate in fully capturing the full outcome of the intervention on patient QoL.

We found that a high percentage of studies that measured QoL used a drug intervention. While it was beyond the scope of this study to estimate the percentage of drug clinical trials that examine QoL, current estimates from prior research indicate that the frequency of patient-reported outcomes are being increasingly used in registered clinical trials.^153^ Guidance by the US Food and Drug Administration encouraging better use of patient-reported outcomes in drug clinical trials and professional organizations in oncology proposing standardized approaches to evaluating clinical trial results may be encouraging progress in the number of drug studies reporting on QoL.

To our knowledge, this is the first study to evaluate the points for when QoL assessments were made in oncology trials. Not only do we report whether studies assessed QoL until death, but the numbers we have presented show that there are large differences in most studies between median survival time and median time to follow-up. Our findings that QoL had positive results in 56% of studies are slightly higher than 1 study^154^ that found that 42% of recently approved oncology drugs improved QoL but are more similar to another study.^155^ The differences may be because of the types of interventions included in the study and the way that QoL outcomes were coded. It is difficult to know whether these results are true to the total population of patients who receive these interventions or if they only apply to people who do well on these drugs. In many studies, QoL is not measured after a patient has progressed, and because no further QoL measurements are assessed, we do not know the subsequent status of their QoL.

A further consideration in oncology studies is that many drugs being tested in clinical trials do not even report on QoL. Recently, it was reported that almost half of drugs for advanced or metastatic solid tumors being tested in phase 3 trials between 2010 and 2015 do not include a QoL outcome, and for those that do, about a quarter of the studies did not report prespecified QoL outcomes.^156^ For drugs approved by the Europeans Medicines Agency (2009-2013) that did not show improvement in overall survival during postmarketing studies, only about 11% showed an improvement in QoL.^155^ Similarly, only 14% of clinical trials registered on ClinicalTrials.gov listed a patient-reported outcome as an outcome of interest.^157^ For the studies we reviewed, only about 7% reported that QoL was a primary outcome. These results collectively suggest the low priority given to QoL assessments, even though most cancer drugs do not improve patient-centered outcomes, such as overall survival,^155,158^ and less than half of approved cancer drugs showed improvement in QoL.^154^ There seems to be discordance between the importance of QoL between researchers and patients, because most patients want to discuss QoL issues with their physicians.^159^

### Limitations

There are several limitations to our work. First, we only examined articles from the 3 highest-impact oncology journals, which may have limited the generalizability of these findings. Similarly, journals may focus on certain types of outcomes, which may bias the results and make them less generalizable. Second, we used the author’s determination of what was considered an appropriate measurement of QoL, and not all QoL metrics measured the same facets of QoL. Most studies used an established survey from either the European Organisation for Research and Treatment or the Functional Assessment of Cancer Therapy, which are widely used, but some instruments were not as well-validated or only focused on functional or emotional facets of QoL. Third, it was not always clear when QoL assessments were done because of insufficient or unclear reporting of methods. To help limit misclassification, at least 2 reviewers and sometimes 3 independently coded QoL assessments. Finally, QoL measurement may not always be reflective of actual QoL, and we were limited to how each study assessed QoL.

## Conclusions

In conclusion, we found that of studies that report on QoL, most assessed QoL during or shortly after the intervention, but few measured QoL until the end of the patient’s life. This is informative because many of the studies that measure QoL until death report worse QoL outcomes for patients in the intervention group, and yet QoL studies with shorter periods measured are increasingly being used for determining health policy decisions.^160^ To justify a therapy’s use based on improved QoL, it is important to show that a therapy improves QoL across the remainder of a patient’s life and not merely while that patient is receiving treatment. Combination or novel therapies may reduce the benefit of salvage medications and lead to worse QoL after progression, negating QoL gains while on therapy, but this would only be known if studies collect QoL during this time. Future research and policy recommendations should consider not just short-term QoL outcomes but QoL outcomes throughout the patient’s life.

## Notes

Supplement. **eAppendix.** Results with references. **eReferences.**
