# Supplementary material for: Patient Experience Captured by Quality-of-Life Measurement in Oncology Clinical Trials
Source: JAMA Netw Open. 2020 Mar 4;3(3):e200363. doi: 10.1001/jamanetworkopen.2020.0363 (PMC7057133; doi:10.1001/jamanetworkopen.2020.0363)
Supplement: Supplement. — eAppendix. Results with references. eReferences. [file jamanetwopen-3-e200363-s001.pdf]

Supplementary Online Content

Haslam A, Herrera-Perez D, Gill J, Prasad V. Patient experience captured by quality-of-life measurement in oncology clinical trials. *JAMA Network Open*. 2020;3(3):e200363. doi:10.1001/jamanetworkopen.2020.0363

eAppendix. Results with references.

eReferences.

This supplementary material has been provided by the authors to give readers additional information about their work.

© 2020 Haslam A et al. *JAMA Network Open*.

eAppendix. Results with references

There were 856 articles reviewed for inclusion, of which 149 met inclusion criteria.[3-151] Studies that were excluded were not RCTs or did not analyze data in randomized groups (544 articles), did not report or assess QoL (123 articles), reported QoL in a separate manuscript (38 articles), was a research letter (1 article), or was a study that combined 3 RCTs (1 article). Seventy-four studies[3-5,7,10,14,15,17-21,23-29,31,32,34,35,39,41,42,45,47,48,50,53-55,59-61,64-70,73,76-78,80,81,86-90,93,94,96-99,101-104,106,109,111,113,117,118,123-125,128] included people with metastatic, advanced, and/or incurable cancers (49.7%); 42 studies[6,8,9,11-13,16,22,30,33,36-38,40,43,44,46,49,51,56,57,62,63,71,72,75,79,82-84,91,92,105,107,114-116,120,122,127,129,130] included patients with cancers that were not metastatic, advanced, or incurable (28.2%); and 33 studies [52,58,74,85,95,100,108,110,112,119,121,126,131-151] included interventions that were not designed to improve survival (22.1%).

Among eligible studies of metastatic, advanced, or incurable cancers (Table 1), 40 studies were published in Lancet Oncology,[3-5,7,10,14,15,17-21,23-29,31-35,39,41,42,45,47,48,50,53-55,59-61,64-67] 31 studies in the Journal of Clinical Oncology,[68-70,73,76-78,80,81,86-90,93,94,96-99,101-104,106,109,111,113,117,118,123] and 3 studies in JAMA Oncology.[124,125,128] Quality of life was the primary study outcome in 2 studies[10,73] (4.1%), whereas most studies did not have QoL as a primary end point (72 articles[3-5,7,14,15,17-21,23-29,31,32,34,35,39,41,42,45,47,48,50,53-55,59-61,64-70,76-78,80,81,86-90,93,94,96-99,101-104,106,109,111,113,117,118,123-125,128] [95.9%]). Most studies used a drug intervention (68 [articles3-5,7,10,14,15,17-21,23-25,27,29,31,32,34,35,39,42,45,47,48,50,53-55,59-61,64,66-68,70,73,76-78,81,86-90,93,94,96-99,101-104,106,109,111,113,117,118,123,125,128] [90.7%]). Forty-four studies[4,5,10,14,20,21,23,24,26,27,29,31,34,35,39,42,45,47,48,50,53-55,59-61,64,67-70,76,77,88,89,94,97,99,101,104,106,109,111,124] (60.0%) reported a positive QoL outcome, 24 studies[7,15,17-19,25,28,32,41,65,66,78,81,86,87,90,96,98,102,103,117,123,125,128] (32.0%) had negative outcomes, and 6 studies[3,73,80,93,113,118] (8.0%) had indeterminate findings.

Among eligible studies with cancers that were not advanced, metastatic, or incurable and studies that used an intervention not designed to improve survival (Table 1), 25 were published in Lancet Oncology,[6,8,9,11-13,16,22,30,33,36-38,40,43,44,46,49,51,52,56-58,62,63] 42 in the Journal of Clinical Oncology,[71-75,79,82-85,91,92,95,100,105,107,108,110,114-116,119-122,131-147] and 8 in JAMA Oncology.[126,127,129,130,148-151] Quality of life was the primary study outcome in 10.8% (9 studies[62,112,132-134,138,140,145,150]), whereas most studies did not have QoL as a primary end point (66 articles[6,8,9,11-13,16,22,30,33,36-38,40,43,44,46,49,51,52,56-58,63,71,72,74,75,79,82-85,91,92,95,100,105,107,108,110,114-116,119-122,126,127,129-131,135-137,139,141-144,146-149,151] [89.2%]). Most studies used a drug intervention (33 articles[11-13,30,33,37,38,43,46,49,51,58,63,72,74,75,79,82,84,85,91,95,100,105,107,110,112,114,115,119-121,126] [44.6%]), 21 studies[131-151] used a behavioral intervention (27.0%), 9 studies[6,16,56,57,71,116,122,129,130] used therapeutic radiation as an intervention (12.2%), 1 study[44] concerned a surgery intervention (1.4%), 8 studies[8,9,22,40,62,83,92,127] used a chemotherapy regimen (with or without surgery, radiation, or another drug [10.8%]), and 3 studies[36,52,108] had some other type of intervention (a device, treatment algorithm, or procedure [4.1%]). The most common QoL outcome was positive (40 articles[6,8,9,11-13,16,33,36,37,43,44,51,57,58,63,71,74,79,82,83,85,95,100,105,116,119,120,133,135,138,140,142,144,145,150,151] [52.7%]); 31 studies[22,38,40,46,52,56,62,72,75,84,91,92,107,108,110,112,114,115,121,127,129,130,134,136,137,139,141,143,146-148] (41.9%) had negative outcomes, and 4 (5.4%) had indeterminate outcomes.[30,49,122,149]

For all studies and interventions, QoL assessment was high during the intervention (66 articles[3-5,10,14,15,17-21,23-25,27-29,31,32,34,39,42,45,48,50,53-55,59,61,64,66-70,73,77,78,81,86-90,93,94,96-99,101-104,106,109,111,113,117,118,123-125,128] [89.2%] on metastatic cancers; 38 articles[8,9,11-13,16,22,33,38,46,49,62,63,72,79,82-84,95,100,107,108,110,112,114,115,119,121,133,134,137,138,145,146,150,151] [50.7%] on nonmetastatic cancers), after the end of the intervention (33 articles[5,7,10,14,17,21,25,28,29,32,35,47,48,60,66,68-70,76-78,86-88,93,99,102,113,117,118,123,125,128] [44.6%] on metastatic cancers; 35 articles[9,30,37,40,49,52,63,74,75,79,82,85,92,95,100,107,110,112,119-122,126,129,131,132,135-137,140,142,143,149,150] [46.7%] on nonmetastatic cancers), and during follow-up (32 articles[3,17,21,23,26-28,34,41,42,45,47,53-

© 2020 Haslam A et al. *JAMA Network Open*.

[55,60,61,65,66,70,73,77,80,81,87,89,90,96,97,99,102,104] [43.2%] on metastatic cancers; 49 articles[6,8,22,30,33,36,38,40,43,44,46,51,52,56-58,62,63,71,72,82-84,91,105,107,114-116,119,120,122,127,129-132,134,135,139-144,146-149] [65.3%] on nonmetastatic cancers) (Table 1). The assessment of QoL until the time of death was low for studies of both metastatic cancers (1 article[41] [1.4%]) and nonmetastatic cancers (4 articles[16,22,114,148] [5.3%]).

For studies that measured QoL during treatment, 87 studies[3-5,7,10-15,17-21,23-25,27-29,31-34,38,39,42,45,46,48-50,53-55,59,61,63,64,66-68,70,72,73,77-79,81,82,84,86-91,93-104,106,107,109-115,117-119,121,123,125,126,128] (83.7%) used a drug intervention and 8 studies[133,134,137,138,145,146,150,151] (7.7%) used a behavioral intervention (Table 2). For studies that measured QoL until the end of treatment, 50 studies[5,7,10,14,17,21,25,28-30,32,35,37,47-49,60,63,66,68,70,72,74-79,85-88,93,95,99,100,102,107,110,112,113,117-121,123,125,126,128] (73.5%) used a drug intervention and 11 studies[131,132,135-137,140,142,143,149-151] (16.2%) used a behavioral intervention. For studies that measured QoL after some amount of follow-up time, 46 studies[3,17,21,23,27,28,30,33,34,38,42,43,45-47,51,53-55,58,60,61,63,66,70,72,73,77,81,82,84,87,89-91,96,97,99,102,104,105,107,114,115,119,120] (56%) used a drug intervention and 14 studies[131,132,134,135,139-144,146-149] (17.3%) used a behavioral intervention. For studies measuring QoL on progression, 25 studies[3,12,14,15,25,38,48,54,59,61,63,66,68,72,76,88,90,94,101,103,106,109,113,123,128] (89.3%) used a drug intervention and none used a behavioral intervention. For studies that measured QoL until death, only 1 study[114] (20%) used a drug intervention, 1 study[148] (20%) used a behavioral intervention, and 2 studies[16,41] (40%) used a radiation intervention.

The number of studies that reported a positive QoL outcome was 59 (56.7%) for studies that measured QoL during treatment[4,5,8-14,16,20,21,23,24,27,29,31,33,34,39,42,45,48,50,53-55,59,61,63,64,67-70,77,79,82,83,88,89,94,95,97,99-101,104,106,109,111,119,124,126,133,138,145,150,151] (Table 3), 35 (51.5%) for studies that measured QoL at the end of treatment,[5,9,10,14,21,29,35,37,47,48,60,63,68-70,74,76,77,79,82,85,88,95,99,100,119,120,126,131,132,135,140,142,150,151] 42 (51.9%) for studies measuring QoL after some amount of follow-up time,[6,8,21,23,26,27,33,34,36,42-45,47,51,53-55,57,58,60,61,63,70,71,77,82,83,89,97,99,104,105,116,119,120,131,132,135,140,142,144] 16 (57.1%) for studies measuring QoL on progression,[12,14,48,54,59,61,63,68,69,71,76,88,94,101,106,109] and 1 (20%) for studies measuring QoL until death.[16] Similar patterns in the distribution of positive QoL outcomes were seen for studies that included metastatic, advanced, or incurable cancers. Figure 1 (for studies in which the median overall survival was reached) and Figure 2 (for studies in which the median overall survival was not reached) show the comparison of overall survival and the duration that QoL was assessed in studies that included patients with metastatic, advanced, or incurable cancers.

© 2020 Haslam A et al. *JAMA Network Open*.

eReferences.

3. Dreno B, Thompson JF, Smithers BM, et al. MAGE-A3 immunotherapeutic as adjuvant therapy for patients with resected, MAGE-A3-positive, stage III melanoma (DERMA): a double-blind, randomised, placebo-controlled, phase 3 trial. Lancet Oncol. 2018;19(7):916-929. doi:10.1016/S1470-2045(18)30254-7

4. Clarke N, Wiechno P, Alekseev B, et al. Olaparib combined with abiraterone in patients with metastatic castration-resistant prostate cancer: a randomised, double-blind, placebo-controlled, phase 2 trial. Lancet Oncol. 2018;19(7):975-986. doi:10.1016/S1470-2045(18)30365-6

5. Tripathy D, Im SA, Colleoni M, et al. Ribociclib plus endocrine therapy for premenopausal women with hormone receptor-positive, advanced breast cancer (MONALEESA-7): a randomised phase 3 trial. Lancet Oncol. 2018;19(7):904-915. doi:10.1016/S1470-2045(18)30292-4

6. Schäfer R, Strnad V, Polgár C, et al; Groupe Européen de Curiethérapie of European Society for Radiotherapy and Oncology (GEC-ESTRO). Quality-of-life results for accelerated partial breast irradiation with interstitial brachytherapy versus whole-breast irradiation in early breast cancer after breast-conserving surgery (GEC-ESTRO): 5-year results of a randomised, phase 3 trial. Lancet Oncol. 2018;19(6):834-844. doi:10.1016/S1470-2045(18)30195-5

7. Rimassa L, Assenat E, Peck-Radosavljevic M, et al. Tivantinib for second-line treatment of MET-high, advanced hepatocellular carcinoma (METIV-HCC): a final analysis of a phase 3, randomised, placebo-controlled study. Lancet Oncol. 2018;19(5):682-693. doi:10.1016/S1470-2045(18)30146-3

8. Iveson TJ, Kerr RS, Saunders MP, et al. 3 versus 6 months of adjuvant oxaliplatin-fluoropyrimidine combination therapy for colorectal cancer (SCOT): an international, randomised, phase 3, non-inferiority trial. Lancet Oncol. 2018;19(4):562-578. doi:10.1016/S1470-2045(18)30093-7

9. Tang L-Q, Chen DP, Guo L, et al. Concurrent chemoradiotherapy with nedaplatin versus cisplatin in stage II-IVB nasopharyngeal carcinoma: an open-label, non-inferiority, randomised phase 3 trial. Lancet Oncol. 2018;19(4):461-473. doi:10.1016/S1470-2045(18)30104-9

10. Chi KN, Protheroe A, Rodríguez-Antolín A, et al. Patient-reported outcomes following abiraterone acetate plus prednisone added to androgen deprivation therapy in patients with newly diagnosed metastatic castration-naïve prostate cancer (LATITUDE): an international, randomised phase 3 trial. Lancet Oncol. 2018;19(2):194-206. doi:10.1016/S1470-2045(17)30911-7

11. Hurvitz SA, Martin M, Symmans WF, et al. Wildiers H, Campone M. Neoadjuvant trastuzumab, pertuzumab, and chemotherapy versus trastuzumab emtansine plus pertuzumab in patients with HER2-positive breast cancer (KRISTINE): a randomised, open-label, multicentre, phase 3 trial. Lancet Oncol. 2018;19(1):115-126.

12. ZhongW-Z, Wang Q, Mao WM, et al; ADJUVANT investigators. Gefitinib versus vinorelbine plus cisplatin as adjuvant treatment for stage II-IIIA (N1-N2) EGFR-mutant NSCLC (ADJUVANT/CTONG1104): a randomised, open label, phase 3 study. Lancet Oncol. 2018;19(1):139-148. doi:10.1016/S1470-2045(17)30729-5

© 2020 Haslam A et al. *JAMA Network Open*.

13. Colleoni M, Luo W, Karlsson P, et al; SOLE Investigators. Extended adjuvant intermittent letrozole versus continuous letrozole in postmenopausal women with breast cancer (SOLE): a multicentre, open-label, randomised, phase 3 trial. Lancet Oncol. 2018;19(1):127-138. doi:10.1016/S1470-2045(17)30715-5

14. Brahmer JR, Rodríguez-Abreu D, Robinson AG, et al. Health-related quality-of-life results for pembrolizumab versus chemotherapy in advanced, PD-L1-positive NSCLC (KEYNOTE-024): a multicentre, international, randomised, open-label phase 3 trial. Lancet Oncol. 2017;18(12):1600-1609. doi:10.1016/S1470-2045(17)30690-3

15. Bang Y-J, Xu RH, Chin K, et al. Olaparib in combination with paclitaxel in patients with advanced gastric cancer who have progressed following first-line therapy (GOLD): a double-blind, randomised, placebo-controlled, phase 3 trial. Lancet Oncol. 2017;18(12):1637-1651. doi:10.1016/S1470-2045(17)30682-4

16. Vilgrain V, Pereira H, Assenat E, et al; SARAH Trial Group. Efficacy and safety of selective internal radiotherapy with yttrium-90 resin microspheres compared with sorafenib in locally advanced and inoperable hepatocellular carcinoma (SARAH): an open-label randomised controlled phase 3 trial. Lancet Oncol. 2017;18(12):1624-1636. doi:10.1016/S1470-2045(17)30683-6

17. Wu Y-L, Cheng Y, Zhou X, et al. Dacomitinib versus gefitinib as first-line treatment for patients with EGFR mutation-positive non-small-cell lung cancer (ARCHER 1050): a randomised, open-label, phase 3 trial. Lancet Oncol. 2017;18(11):1454-1466. doi:10.1016/S1470-2045(17)30608-3

18. Seddon B, Strauss SJ, Whelan J, et al. Gemcitabine and docetaxel versus doxorubicin as first-line treatment in previously untreated advanced unresectable or metastatic soft-tissue sarcomas (GeDDiS): a randomized controlled phase 3 trial. Lancet Oncol. 2017;18(10):1397-1410. doi:10.1016/S1470-2045(17)30622-8

19. Weller M, Butowski N, Tran DD, et al; ACT IV trial investigators. Rindopepimut with temozolomide for patients with newly diagnosed, EGFRvIII-expressing glioblastoma (ACT IV): a randomised, double-blind, international phase 3 trial. Lancet Oncol. 2017;18(10):1373-1385. doi:10.1016/S1470-2045(17)30517-X

20. Pavel ME, Singh S, Strosberg JR, et al. Health-related quality of life for everolimus versus placebo in patients with advanced, non-functional, well-differentiated gastrointestinal or lung neuroendocrine tumours (RADIANT-4): a multicentre, randomised, double-blind, placebo-controlled, phase 3 trial. Lancet Oncol. 2017;18(10):1411-1422. doi:10.1016/S1470-2045(17)30471-0

21. Kim S-B, Dent R, Im SA, et al; LOTUS investigators. Ipatasertib plus paclitaxel versus placebo plus paclitaxel as first-line therapy for metastatic triple-negative breast cancer (LOTUS): a multicentre, randomised, double-blind, placebo-controlled, phase 2 trial. Lancet Oncol. 2017;18(10):1360-1372. doi:10.1016/S1470-2045(17)30450-3

22. Alderson D, Cunningham D, Nankivell M, et al. Neoadjuvant cisplatin and fluorouracil versus epirubicin, cisplatin, and capecitabine followed by resection in patients with oesophageal adenocarcinoma (UK MRC OE05): an open-label, randomised phase 3 trial. Lancet Oncol. 2017;18(9):1249-1260. doi:10.1016/S1470-2045(17)30447-3

© 2020 Haslam A et al. *JAMA Network Open*.

23. Duchesne GM, Woo HH, King M, et al. Health-related quality of life for immediate versus delayed androgen deprivation therapy in patients with asymptomatic, non-curable prostate cancer (TROG 03.06 and VCOG PR 01-03 [TOAD]): a randomised, multicentre, non-blinded, phase 3 trial. Lancet Oncol. 2017;18(9):1192-1201. doi:10.1016/S1470-2045(17)30426-6

24. Pujade-Lauraine E, Ledermann JA, Selle F, et al; SOLO2/ENGOT-Ov21 investigators. Olaparib tablets as maintenance therapy in patients with platinum-sensitive, relapsed ovarian cancer and a BRCA1/2 mutation (SOLO2/ENGOT-Ov21): a double-blind, randomised, placebo-controlled, phase 3 trial. Lancet Oncol. 2017;18(9):1274-1284. doi:10.1016/S1470-2045(17)30469-2

25. Maio M, Scherpereel A, Calabrò L, et al. Tremelimumab as second-line or third-line treatment in relapsed malignant mesothelioma (DETERMINE): a multicentre, international, randomised, double-blind, placebo controlled phase 2b trial. Lancet Oncol. 2017;18(9):1261-1273. doi:10.1016/S1470-2045(17)30446-1

26. Brown PD, Ballman KV, Cerhan JH, et al. Postoperative stereotactic radiosurgery compared with whole brain radiotherapy for resected metastatic brain disease (NCCTG N107C/CEC·3): a multicentre, randomised, controlled, phase 3 trial. Lancet Oncol. 2017;18(8):1049-1060. doi:10.1016/S1470-2045(17)30441-2

27. Harrington KJ, Ferris RL, Blumenschein G Jr, et al. Nivolumab versus standard, single-agent therapy of investigator's choice in recurrent or metastatic squamous cell carcinoma of the head and neck (CheckMate 141): health-related quality-of-life results from a randomised, phase 3 trial. Lancet Oncol. 2017;18(8):1104-1115. doi:10.1016/S1470-2045(17)30421-7

28. Tap WD, Papai Z, Van Tine BA, et al. Doxorubicin plus evofosfamide versus doxorubicin alone in locally advanced, unresectable or metastatic soft-tissue sarcoma (TH CR-406/SARC021): an international, multicentre, open-label, randomised phase 3 trial. Lancet Oncol. 2017;18(8):1089-1103. doi:10.1016/S1470-2045(17)30381-9

29. Shaw AT, Kim TM, Crinò L, et al. Ceritinib versus chemotherapy in patients with ALK-rearranged non-small-cell lung cancer previously given chemotherapy and crizotinib (ASCEND-5): a randomised, controlled, open-label, phase 3 trial. Lancet Oncol. 2017;18(7):874-886. doi:10.1016/S1470-2045(17)30339-X

30. Cameron D, Morden JP, Canney P, et al; TACT2 Investigators. Accelerated versus standard epirubicin followed by cyclophosphamide, methotrexate, and fluorouracil or capecitabine as adjuvant therapy for breast cancer in the randomised UK TACT2 trial (CRUK/05/19): a multicentre, phase 3, open-label, randomised, controlled trial. Lancet Oncol. 2017;18(7):929-945. doi:10.1016/S1470-2045(17)30404-7

31. Coleman RL, Brady MF, Herzog TJ, et al. Bevacizumab and paclitaxel-carboplatin chemotherapy and secondary cytoreduction in recurrent, platinum-sensitive ovarian cancer (NRG Oncology/Gynecologic Oncology Group study GOG-0213): a multicentre, open-label, randomised, phase 3 trial. Lancet Oncol. 2017;18(6):779-791. doi:10.1016/S1470-2045(17)30279-6

© 2020 Haslam A et al. *JAMA Network Open*.

32. Ascierto PA, Del Vecchio M, Robert C, et al. Ipilimumab 10mg/kg versus ipilimumab 3mg/kg in patients with unresectable or metastatic melanoma: a randomised, double-blind, multicentre, phase 3 trial. Lancet Oncol. 2017;18(5):611-622. doi:10.1016/S1470-2045(17)30231-0

33. Coens C, Suciu S, Chiarion-Sileni V, et al. Health-related quality of life with adjuvant ipilimumab versus placebo after complete resection of high-risk stage III melanoma (EORTC 18071): secondary outcomes of a multinational, randomised, double-blind, phase 3 trial. Lancet Oncol. 2017;18(3):393-403. doi:10.1016/S1470-2045(17)30015-3

34. Soulières D, Faivre S, Mesía R, et al. Buparlisib and paclitaxel in patients with platinum-pretreated recurrent or metastatic squamous cell carcinoma of the head and neck (BERIL-1): a randomised, double-blind, placebo controlled phase 2 trial. Lancet Oncol. 2017;18(3):323-335. doi:10.1016/S1470-2045(17)30064-5

35. Hickish T, Andre T, Wyrwicz L, et al. MABp1 as a novel antibody treatment for advanced colorectal cancer: a randomised, double-blind, placebo-controlled, phase 3 study. Lancet Oncol. 2017;18(2):192-201. doi:10.1016/S1470-2045(17)30006-2

36. Azzouzi A-R, Vincendeau S, Barret E, et al; PCM301 Study Group. Padeliporfin vascular-targeted photodynamic therapy versus active surveillance in men with low-risk prostate cancer (CLIN1001 PCM301): an open-label, phase 3, randomised controlled trial. Lancet Oncol. 2017;18(2):181-191. doi:10.1016/S1470-2045(16)30661-1

37. Passamonti F, Griesshammer M, Palandri F, et al. Ruxolitinib for the treatment of inadequately controlled polycythaemia vera without splenomegaly (RESPONSE-2): a randomised, open-label, phase 3b study. Lancet Oncol. 2017;18(1):88-99. doi:10.1016/S1470-2045(16)30558-7

38. Reijneveld JC, Taphoorn MJB, Coens C, et al. Health-related quality of life in patients with high-risk low-grade glioma (EORTC 22033-26033): a randomised, open-label, phase 3 intergroup study. Lancet Oncol. 2016;17(11):1533-1542. doi:10.1016/S1470-2045(16)30305-9

39. Ascierto PA, McArthur GA, Dréno B, et al. Cobimetinib combined with vemurafenib in advanced BRAF(V600)- mutant melanoma (coBRIM): updated efficacy results from a randomised, double-blind, phase 3 trial. Lancet Oncol. 2016;17(9):1248-1260. doi:10.1016/S1470-2045(16)30122-X

40. de Boer SM, Powell ME, Mileshkin L, et al; PORTEC study group. Toxicity and quality of life after adjuvant chemoradiotherapy versus radiotherapy alone for women with high-risk endometrial cancer (PORTEC-3): an open label, multicentre, randomised, phase 3 trial. Lancet Oncol. 2016;17(8):1114-1126. doi:10.1016/S1470-2045(16)30120-6

41. Clive AO, Taylor H, Dobson L, et al. Prophylactic radiotherapy for the prevention of procedure-tract metastases after surgical and large-bore pleural procedures in malignant pleural mesothelioma (SMART): a multicentre, open-label, phase 3, randomised controlled trial. Lancet Oncol. 2016;17(8):1094-1104. doi:10.1016/S1470-2045(16)30095-X

© 2020 Haslam A et al. *JAMA Network Open*.

42. Cella D, Grünwald V, Nathan P, et al. Quality of life in patients with advanced renal cell carcinoma given nivolumab versus everolimus in CheckMate 025: a randomised, open-label, phase 3 trial. Lancet Oncol. 2016;17(7):994-1003. doi:10.1016/S1470-2045(16)30125-5

43. Carrie C, Hasbini A, de Laroche G, et al. Salvage radiotherapy with or without short-term hormone therapy for rising prostate-specific antigen concentration after radical prostatectomy (GETUG-AFU 16): a randomised, multicentre, open-label phase 3 trial. Lancet Oncol. 2016;17(6):747-756. doi:10.1016/S1470-2045(16)00111-X

44. Bendixen M, Jørgensen OD, Kronborg C, Andersen C, Licht PB. Postoperative pain and quality of life after lobectomy via video-assisted thoracoscopic surgery or anterolateral thoracotomy for early stage lung cancer: a randomised controlled trial. Lancet Oncol. 2016;17(6):836-844. doi:10.1016/S1470-2045(16)00173-X

45. Duchesne GM, Woo HH, Bassett JK, et al. Timing of androgen-deprivation therapy in patients with prostate cancer with a rising PSA (TROG 03.06 and VCOG PR 01-03 [TOAD]): a randomised, multicentre, non-blinded, phase 3 trial. Lancet Oncol. 2016;17(6):727-737. doi:10.1016/S1470-2045(16)00107-8

46. Vansteenkiste JF, Cho BC, Vanakesa T, et al. Efficacy of the MAGE-A3 cancer immunotherapeutic as adjuvant therapy in patients with resected MAGE-A3-positive non-small-cell lung cancer (MAGRIT): a randomised, double blind, placebo-controlled, phase 3 trial. Lancet Oncol. 2016;17(6):822-835. doi:10.1016/S1470-2045(16)00099-1

47. Park K, Tan EH, O'Byrne K, et al. Afatinib versus gefitinib as first-line treatment of patients with EGFR mutation-positive non-small-cell lung cancer (LUX-Lung 7): a phase 2B, open-label, randomised controlled trial. Lancet Oncol. 2016;17(5):577-589. doi:10.1016/S1470-2045(16)30033-X

48. Trněný M, Lamy T, Walewski J, et al; SPRINT trial investigators and in collaboration with the European Mantle Cell Lymphoma Network. Lenalidomide versus investigator's choice in relapsed or refractory mantle cell lymphoma (MCL-002; SPRINT): a phase 2, randomised, multicentre trial. Lancet Oncol. 2016;17(3):319-331. doi:10.1016/S1470-2045(15)00559-8

49. Chan A, Delaloge S, Holmes FA, et al; ExteNET Study Group. Neratinib after trastuzumab-based adjuvant therapy in patients with HER2-positive breast cancer (ExteNET): a multicentre, randomised, double-blind, placebo-controlled, phase 3 trial. Lancet Oncol. 2016;17(3):367-377. doi:10.1016/S1470-2045(15)00551-3

50. Armstrong AJ, Halabi S, Eisen T, et al. Everolimus versus sunitinib for patients with metastatic non-clear cell renal cell carcinoma (ASPEN): a multicentre, open-label, randomised phase 2 trial. Lancet Oncol. 2016;17(3):378-388. doi:10.1016/S1470-2045(15)00515-X

51. Walker I, Panzarella T, Couban S, et al; Canadian Blood and Marrow Transplant Group. Pretreatment with anti-thymocyte globulin versus no anti-thymocyte globulin in patients with haematological malignancies undergoing haemopoietic cell transplantation from unrelated donors: a randomised, controlled, open-label, phase 3, multicentre trial. Lancet Oncol. 2016;17(2):164-173. doi:10.1016/S1470-2045(15)00462-3

© 2020 Haslam A et al. *JAMA Network Open*.

52. Glover M, Smerdon GR, Andreyev HJ, et al. Hyperbaric oxygen for patients with chronic bowel dysfunction after pelvic radiotherapy (HOT2): a randomised, double-blind, sham-controlled phase 3 trial. Lancet Oncol. 2016;17(2):224-233. doi:10.1016/S1470-2045(15)00461-1

53. Takashima T, Mukai H, Hara F, et al; SELECT BC Study Group. Taxanes versus S-1 as the first-line chemotherapy for metastatic breast cancer (SELECT BC): an open-label, non-inferiority, randomised phase 3 trial. Lancet Oncol. 2016;17(1):90-98. doi:10.1016/S1470-2045(15)00411-8

54. du Bois A, Kristensen G, Ray-Coquard I, et al; AGO Study Group led Gynecologic Cancer Intergroup/European Network of Gynaecologic Oncology Trials Groups Intergroup Consortium. Standard first-line chemotherapy with or without nintedanib for advanced ovarian cancer (AGO-OVAR 12): a randomised, double-blind, placebo controlled phase 3 trial. Lancet Oncol. 2016;17(1):78-89. doi:10.1016/S1470-2045(15)00366-6

55. Place AE, Stevenson KE, Vrooman LM, et al. Intravenous pegylated asparaginase versus intramuscular native Escherichia coli L-asparaginase in newly diagnosed childhood acute lymphoblastic leukaemia (DFCI 05-001): a randomised, open-label phase 3 trial. Lancet Oncol. 2015;16(16):1677-1690. doi:10.1016/S1470-2045(15)00363-0

56. Stahel RA, Riesterer O, Xyrafas A, et al. Neoadjuvant chemotherapy and extrapleural pneumonectomy of malignant pleural mesothelioma with or without hemithoracic radiotherapy (SAKK 17/04): a randomised, international, multicentre phase 2 trial. Lancet Oncol. 2015;16(16):1651-1658. doi:10.1016/S1470-2045(15)00208-9

57. Wilkins A, Mossop H, Syndikus I, et al. Hypofractionated radiotherapy versus conventionally fractionated radiotherapy for patients with intermediate-risk localised prostate cancer: 2-year patient-reported outcomes of the randomised, non-inferiority, phase 3 CHHiP trial. Lancet Oncol. 2015;16(16):1605-1616. doi:10.1016/S1470-2045(15)00280-6

58. Chow E, Meyer RM, Ding K, et al. Dexamethasone in the prophylaxis of radiation-induced pain flare after palliative radiotherapy for bone metastases: a double-blind, randomised placebo-controlled, phase 3 trial. Lancet Oncol. 2015;16(15):1463-1472. doi:10.1016/S1470-2045(15)00199-0

59. Perez EA, Awada A, O'Shaughnessy J, et al. Etirinotecan pegol (NKTR-102) versus treatment of physician's choice in women with advanced breast cancer previously treated with an anthracycline, a taxane, and capecitabine (BEACON): a randomised, open-label, multicentre, phase 3 trial. Lancet Oncol. 2015;16(15):1556-1568. doi:10.1016/S1470-2045(15)00332-0

60. Symonds RP, Gourley C, Davidson S, et al. Cediranib combined with carboplatin and paclitaxel in patients with metastatic or recurrent cervical cancer (CIRCCa): a randomised, double-blind, placebo-controlled phase 2 trial. Lancet Oncol. 2015;16(15):1515-1524. doi:10.1016/S1470-2045(15)00220-X

61. Grob JJ, Amonkar MM, Karaszewska B, et al. Comparison of dabrafenib and trametinib combination therapy with vemurafenib monotherapy on health-related quality of life in patients with unresectable or metastatic cutaneous BRAF Val600-mutation-positive melanoma (COMBI-v): results of a phase 3, open-label, randomized trial. Lancet Oncol. 2015;16(13):1389-1398. doi:10.1016/S1470-2045(15)00087-X

© 2020 Haslam A et al. *JAMA Network Open*.

62. Burnett AK, Russell NH, Hills RK, et al; UK National Cancer Research Institute Acute Myeloid Leukaemia Working Group. Arsenic trioxide and all-trans retinoic acid treatment for acute promyelocytic leukaemia in all risk groups (AML17): results of a randomised, controlled, phase 3 trial. Lancet Oncol. 2015;16(13):1295-1305. doi:10.1016/S1470-2045(15)00193-X

63. van Oers MHJ, Kuliczkowski K, Smolej L, et al; PROLONG study investigators. Ofatumumab maintenance versus observation in relapsed chronic lymphocytic leukaemia (PROLONG): an open-label, multicentre, randomised phase 3 study. Lancet Oncol. 2015;16(13):1370-1379. doi:10.1016/S1470-2045(15)00143-6

64. Hegewisch-Becker S, Graeven U, Lerchenmüller CA, et al. Maintenance strategies after first-line oxaliplatin plus fluoropyrimidine plus bevacizumab for patients with metastatic colorectal cancer (AIO 0207): a randomised, non-inferiority, open-label, phase 3 trial. Lancet Oncol. 2015;16(13):1355-1369. doi:10.1016/S1470-2045(15)00042-X

65. Henderson MA, Burmeister BH, Ainslie J, et al. Adjuvant lymph-node field radiotherapy versus observation only in patients with melanoma at high risk of further lymph-node field relapse after lymphadenectomy (ANZMTG 01.02/TROG 02.01): 6-year follow-up of a phase 3, randomised controlled trial. Lancet Oncol. 2015;16(9):1049-1060. doi:10.1016/S1470-2045(15)00187-4

66. Soria J-C, Wu YL, Nakagawa K, et al. Gefitinib plus chemotherapy versus placebo plus chemotherapy in EGFR mutation-positive non-small-cell lung cancer after progression on first-line gefitinib (IMPRESS): a phase 3 randomised trial. Lancet Oncol. 2015;16(8):990-998. doi:10.1016/S1470-2045(15)00121-7

67. Soria J-C, Felip E, Cobo M, et al; LUX-Lung 8 Investigators. Afatinib versus erlotinib as second-line treatment of patients with advanced squamous cell carcinoma of the lung (LUX-Lung 8): an open-label randomized controlled phase 3 trial. Lancet Oncol. 2015;16(8):897-907. doi:10.1016/S1470-2045(15)00006-6

68. Lee CK, Novello S, Rydén A, Mann H, Mok T. Patient-reported symptoms and impact of treatment with osimertinib versus chemotherapy in advanced non-small-cell lung cancer: the AURA3 trial. J Clin Oncol. 2018;36 (18):1853-1860. doi:10.1200/JCO.2017.77.2293

69. Strosberg J, Wolin E, Chasen B, et al; NETTER-1 Study Group. Health-related quality of life in patients with progressive midgut neuroendocrine tumors treated with 177Lu-dotatate in the phase III NETTER-1 trial. J Clin Oncol. 2018;36(25):2578-2584. doi:10.1200/JCO.2018.78.5865

70. Vaughn DJ, Bellmunt J, Fradet Y, et al. Health-related quality-of-life analysis from KEYNOTE-045: a phase III study of pembrolizumab versus chemotherapy for previously treated advanced urothelial cancer. J Clin Oncol. 2018;36(16):1579-1587. doi:10.1200/JCO.2017.76.9562

71. De Ruysscher D, Dingemans AC, Praag J, et al. Prophylactic cranial irradiation versus observation in radically treated stage III non-small-cell lung cancer: a randomized phase III NVALT-11/DLCRG-02 study. J Clin Oncol. 2018;36(23):2366-2377. doi:10.1200/JCO.2017.77.5817

72. Porceddu SV, Bressel M, Poulsen MG, et al. Postoperative concurrent chemoradiotherapy versus postoperative radiotherapy in high-risk cutaneous squamous cell carcinoma of the head and neck: the

© 2020 Haslam A et al. *JAMA Network Open*.

randomized phase III TROG 05.01 trial. J Clin Oncol. 2018;36(13):1275-1283. doi:10.1200/JCO.2017.77.0941

73. Morgans AK, Chen YH, Sweeney CJ, et al. Quality of life during treatment with chemohormonal therapy: analysis of E3805 chemohormonal androgen ablation randomized trial in prostate cancer. J Clin Oncol. 2018;36(11):1088-1095. doi:10.1200/JCO.2017.75.3335

74. Ito Y, Tsuda T, Minatogawa H, et al. Placebo-controlled, double-blinded phase iii study comparing dexamethasone on day 1 with dexamethasone on days 1 to 3 with combined neurokinin-1 receptor antagonist and palonosetron in high-emetogenic chemotherapy. J Clin Oncol. 2018;36(10):1000-1006. doi:10.1200/JCO.2017.74.4375

75. Grill J, Massimino M, Bouffet E, et al. Phase II, open-label, randomized, multicenter trial (HERBY) of bevacizumab in pediatric patients with newly diagnosed high-grade glioma. J Clin Oncol. 2018;36(10):951-958. doi:10.1200/JCO.2017.76.0611

76. Johnston SRD, Hegg R, Im SA, et al. Phase III, randomized study of dual human epidermal growth factor receptor 2 (HER2) blockade with lapatinib plus trastuzumab in combination with an aromatase inhibitor in postmenopausal women with HER2-positive, hormone receptor-positive metastatic breast cancer: ALTERNATIVE. J Clin Oncol. 2018;36(8):741-748. doi:10.1200/JCO.2017.74.7824

77. Cella D, Escudier B, Tannir NM, et al. Quality of life outcomes for cabozantinib versus everolimus in patients with metastatic renal cell carcinoma: METEOR phase III randomized trial. J Clin Oncol. 2018;36(8):757-764. doi:10.1200/JCO.2017.75.2170

78. Aparicio T, Ghiringhelli F, Boige V, et al; PRODIGE 9 Investigators. Bevacizumab maintenance versus no maintenance during chemotherapy-free intervals in metastatic colorectal cancer: a randomized phase III trial (PRODIGE 9). J Clin Oncol. 2018;36(7):674-681. doi:10.1200/JCO.2017.75.2931

79. Lemieux J, Brundage MD, Parulekar WR, et al. Quality of life from Canadian Cancer Trials Group MA.17R: a randomized trial of extending adjuvant letrozole to 10 years. J Clin Oncol. 2018;36(6):563-571. doi:10.1200/JCO.2017.75.7500

80. Ost P, Reynders D, Decaestecker K, et al. Surveillance or metastasis-directed therapy for oligometastatic prostate cancer recurrence: a prospective, randomized, multicenter phase II trial. J Clin Oncol. 2018;36(5):446-453. doi:10.1200/JCO.2017.75.4853

81. Larkin J, Minor D, D'Angelo S, et al. Overall survival in patients with advanced melanoma who received nivolumab versus investigator's choice chemotherapy in CheckMate 037: a randomized, controlled, open-label phase III trial. J Clin Oncol. 2018;36(4):383-390. doi:10.1200/JCO.2016.71.8023

82. Henry NL, Unger JM, Schott AF, et al. Randomized, multicenter, placebo-controlled clinical trial of duloxetine versus placebo for aromatase inhibitor-associated arthralgias in early-stage breast cancer: SWOG S1202. J Clin Oncol. 2018;36(4):326-332. doi:10.1200/JCO.2017.74.6651

© 2020 Haslam A et al. *JAMA Network Open*.

83. Noordman BJ, Verdam MGE, Lagarde SM, et al. Effect of neoadjuvant chemoradiotherapy on health-related quality of life in esophageal or junctional cancer: results from the randomized CROSS trial. J Clin Oncol. 2018;36 (3):268-275. doi:10.1200/JCO.2017.73.7718

84. Motzer RJ, Haas NB, Donskov F, et al; PROTECT investigators. Randomized phase III trial of adjuvant pazopanib versus placebo after nephrectomy in patients with localized or locally advanced renal cell carcinoma. J Clin Oncol. 2017;35(35):3916-3923. doi:10.1200/JCO.2017.73.5324

85. Zhang L, Qu X, Teng Y, et al. Efficacy of thalidomide in preventing delayed nausea and vomiting induced by highly emetogenic chemotherapy: a randomized, multicenter, double-blind, placebo-controlled phase III trial (CLOG1302 study). J Clin Oncol. 2017;35(31):3558-3565. doi:10.1200/JCO.2017.72.2538

86. Pignata S, Scambia G, Bologna A, et al. Randomized controlled trial testing the efficacy of platinum-free interval prolongation in advanced ovarian cancer: the MITO-8, MaNGO, BGOG-Ov1, AGO-Ovar2.16, ENGOT-Ov1, GCIG study. J Clin Oncol. 2017;35(29):3347-3353. doi:10.1200/JCO.2017.73.4293

87. Oudard S, Fizazi K, Sengeløv L, et al. Cabazitaxel versus docetaxel as first-line therapy for patients with metastatic castration-resistant prostate cancer: a randomized phase III trial—FIRSTANA. J Clin Oncol. 2017;35(28):3189-3197. doi:10.1200/JCO.2016.72.1068

88. Eisenberger M, Hardy-Bessard AC, Kim CS, et al; Phase III Study Comparing a Reduced Dose of Cabazitaxel. Phase III study comparing a reduced dose of cabazitaxel (20mg/m2) and the currently approved dose (25mg/m2) in post-docetaxel patients with metastatic castration-resistant prostate cancer—PROSELICA. J Clin Oncol. 2017;35(28):3198-3206. doi:10.1200/JCO.2016.72.1076

89. Kim D-W, Tiseo M, Ahn MJ, et al. Brigatinib in patients with crizotinib-refractory anaplastic lymphoma kinase positive non-small-cell lung cancer: a randomized, multicenter phase II trial. J Clin Oncol. 2017;35(22): 2490-2498. doi:10.1200/JCO.2016.71.5904

90. Jones RJ, Hussain SA, Protheroe AS, et al. Randomized phase II study investigating pazopanib versus weekly paclitaxel in relapsed or progressive urothelial cancer. J Clin Oncol. 2017;35(16):1770-1777. doi:10.1200/JCO.2016.70.7828

91. Agarwala SS, Lee SJ, Yip W, et al. Phase III randomized study of 4 weeks of high-dose interferon-α-2b in stage T2bNO, T3a-bNO, T4a-bNO, and T1-4N1a-2a (microscopic) melanoma: a trial of the Eastern Cooperative Oncology Group-American College of Radiology Imaging Network Cancer Research Group (E1697). J Clin Oncol. 2017;35(8):885-892. doi:10.1200/JCO.2016.70.2951

92. Platzbecker U, Avvisati G, Cicconi L, et al. Improved outcomes with retinoic acid and arsenic trioxide compared with retinoic acid and chemotherapy in non-high-risk acute promyelocytic leukemia: final results of the randomized Italian-German APL0406 trial. J Clin Oncol. 2017;35(6):605-612. doi:10.1200/JCO.2016.67.1982

93. Lee SM, Falzon M, Blackhall F, et al. Randomized prospective biomarker trial of ercc1 for comparing platinum and nonplatinum therapy in advanced non-small-cell lung cancer: ERCC1 trial (ET). J Clin Oncol. 2017;35(4):402-411. doi:10.1200/JCO.2016.68.1841

© 2020 Haslam A et al. *JAMA Network Open*.

94. Perez EA, Barrios C, Eiermann W, et al. Trastuzumab emtansine with or without pertuzumab versus trastuzumab plus taxane for human epidermal growth factor receptor 2-positive, advanced breast cancer: primary results from the phase III MARIANNE study. J Clin Oncol. 2017;35(2):141-148. doi:10.1200/JCO.2016.67.4887

95. Kulke MH, Hörsch D, Caplin ME, et al. Telotristat ethyl, a tryptophan hydroxylase inhibitor for the treatment of carcinoid syndrome. J Clin Oncol. 2017;35(1):14-23. doi:10.1200/JCO.2016.69.2780

96. Liu JF, Ray-Coquard I, Selle F, et al. Randomized phase II trial of seribantumab in combination with paclitaxel in patients with advanced platinum-resistant or -refractory ovarian cancer. J Clin Oncol. 2016;34(36):4345-4353. doi:10.1200/JCO.2016.67.1891

97. Stewart AK, Dimopoulos MA, Masszi T, et al. Health-related quality-of-life results from the open-label, randomized, phase III ASPIRE trial evaluating carfilzomib, lenalidomide, and dexamethasone versus lenalidomide and dexamethasone in patients with relapsed multiple myeloma. J Clin Oncol. 2016;34(32):3921-3930. doi:10.1200/JCO.2016.66.9648

98. Gill S, Ko YJ, Cripps C, et al. PANCREOX: a randomized phase III study of fluorouracil/leucovorin with or without oxaliplatin for second-line advanced pancreatic cancer in patients who have received gemcitabine-based chemotherapy. J Clin Oncol. 2016;34(32):3914-3920. doi:10.1200/JCO.2016.68.5776

99. Hulin C, Belch A, Shustik C, et al. Updated outcomes and impact of age with lenalidomide and low-dose dexamethasone or melphalan, prednisone, and thalidomide in the randomized, phase III FIRST trial. J Clin Oncol. 2016;34(30):3609-3617. doi:10.1200/JCO.2016.66.7295

100. Santini V, Almeida A, Giagounidis A, et al. Randomized phase III study of lenalidomide versus placebo in RBC transfusion-dependent patients with lower-risk Non-del(5q) myelodysplastic syndromes and ineligible for or refractory to erythropoiesis-stimulating agents. J Clin Oncol. 2016;34(25):2988-2996. doi:10.1200/JCO.2015.66.0118

101. Pavlakis N, Sjoquist KM, Martin AJ, et al. Regorafenib for the treatment of advanced gastric cancer (INTEGRATE): a multinational placebo-controlled phase II trial. J Clin Oncol. 2016;34(23):2728-2735. doi:10.1200/JCO.2015.65.1901

102. Sternberg C, Armstrong A, Pili R, et al. Randomized, double-blind, placebo-controlled phase III study of tasquinimod in men with metastatic castration-resistant prostate cancer. J Clin Oncol. 2016;34(22):2636-2643. doi:10.1200/JCO.2016.66.9697

103. Segelov E, Thavaneswaran S, Waring PM, et al. Response to cetuximab with or without irinotecan in patients with refractory metastatic colorectal cancer harboring the KRAS G13D mutation: Australasian Gastro-Intestinal Trials Group ICECREAM study. J Clin Oncol. 2016;34(19):2258-2264. doi:10.1200/JCO.2015.65.6843

104. Penson DF, Armstrong AJ, Concepcion R, et al. Enzalutamide versus bicalutamide in castration-resistant prostate cancer: the STRIVE trial. J Clin Oncol. 2016;34(18):2098-2106. doi:10.1200/JCO.2015.64.9285

© 2020 Haslam A et al. *JAMA Network Open*.

105. Bolla M, Maingon P, Carrie C, et al. Short androgen suppression and radiation dose escalation for intermediate- and high-risk localized prostate cancer: results of EORTC trial 22991. J Clin Oncol. 2016;34(15):1748-1756. doi:10.1200/JCO.2015.64.8055

106. Herrlinger U, Schäfer N, Steinbach JP, et al. Bevacizumab plus irinotecan versus temozolomide in newly diagnosed O6-methylguanine-DNA methyltransferase nonmethylated glioblastoma: the randomized GLARIUS trial. J Clin Oncol. 2016;34(14):1611-1619. doi:10.1200/JCO.2015.63.4691

107. Ribi K, Luo W, Bernhard J, et al. Adjuvant tamoxifen plus ovarian function suppression versus tamoxifen alone in premenopausal women with early breast cancer: patient-reported outcomes in the Suppression of Ovarian Function trial. J Clin Oncol. 2016;34(14):1601-1610. doi:10.1200/JCO.2015.64.8675

108. Corre R, Greillier L, Le Caër H, et al. Use of a comprehensive geriatric assessment for the management of elderly patients with advanced non-small-cell lung cancer: the phase III randomized ESOGIA-GFPC-GECP 08-02 study. J Clin Oncol. 2016;34(13):1476-1483. doi:10.1200/JCO.2015.63.5839

109. Li J, Qin S, Xu J, et al. Randomized, double-blind, placebo-controlled phase III trial of apatinib in patients with chemotherapy-refractory advanced or metastatic adenocarcinoma of the stomach or gastroesophageal junction. J Clin Oncol. 2016;34(13):1448-1454. doi:10.1200/JCO.2015.63.5995

110. Melosky B, Anderson H, Burkes RL, et al. Pan Canadian rash trial: a randomized phase III trial evaluating the impact of a prophylactic skin treatment regimen on epidermal growth factor receptor-tyrosine kinase inhibitor induced skin toxicities in patients with metastatic lung cancer. J Clin Oncol. 2016;34(8):810-815. doi:10.1200/JCO.2015.62.3918

111. Pujade-Lauraine E, Selle F, Weber B, et al. Volasertib versus chemotherapy in platinum-resistant or –refractory ovarian cancer: a randomized phase II Groupe des Investigateurs Nationaux pour l'Etude des Cancers de l'Ovaire Study. J Clin Oncol. 2016;34(7):706-713. doi:10.1200/JCO.2015.62.1474

112. Fallon M, Hoskin PJ, Colvin LA, et al. Randomized double-blind trial of pregabalin versus placebo in conjunction with palliative radiotherapy for cancer-induced bone pain. J Clin Oncol. 2016;34(6):550-556. doi:10.1200/JCO.2015.63.8221

113. Hecht JR, Bang YJ, Qin SK, et al. Lapatinib in combination with capecitabine plus oxaliplatin in human epidermal growth factor receptor 2-positive advanced or metastatic gastric, esophageal, or gastroesophageal adenocarcinoma: TRIO-013/LOGiC—a randomized phase III trial. J Clin Oncol. 2016;34(5):443-451. doi:10.1200/JCO.2015.62.6598

114. Macbeth F, Noble S, Evans J, et al. Randomized phase III trial of standard therapy plus low molecular weight heparin in patients with lung cancer: FRAGMATIC trial. J Clin Oncol. 2016;34(5):488-494. doi:10.1200/JCO.2015.64.0268

115. Harrington K, Temam S, Mehanna H, et al. Postoperative adjuvant lapatinib and concurrent chemoradiotherapy followed by maintenance lapatinib monotherapy in high-risk patients with resected squamous cell carcinoma of the head and neck: a phase III, randomized, double-blind, placebo-controlled study. J Clin Oncol. 2015;33(35):4202-4209. doi:10.1200/JCO.2015.61.4370

© 2020 Haslam A et al. *JAMA Network Open*.

116. Ghadjar P, Hayoz S, Bernhard J, et al. Acute toxicity and quality of life after dose-intensified salvage radiation therapy for biochemically recurrent prostate cancer after prostatectomy: first results of the randomized trial SAKK 09/10. J Clin Oncol. 2015;33(35):4158-4166. doi:10.1200/JCO.2015.63.3529

117. Mohr P, Hauschild A, Trefzer U, et al. Intermittent high-dose intravenous interferon alfa-2b for adjuvant treatment of stage III melanoma: final analysis of a randomized phase III Dermatologic Cooperative Oncology Group trial. J Clin Oncol. 2015;33(34):4077-4084. doi:10.1200/JCO.2014.59.6932

118. Hurwitz HI, Uppal N, Wagner SA, et al. Randomized, double-blind, phase II study of ruxolitinib or placebo in combination with capecitabine in patients with metastatic pancreatic cancer for whom therapy with gemcitabine has failed. J Clin Oncol. 2015;33(34):4039-4047. doi:10.1200/JCO.2015.61.4578

119. Goetsch MF, Lim JY, Caughey AB. A practical solution for dyspareunia in breast cancer survivors: a randomized controlled trial. J Clin Oncol. 2015;33(30):3394-3400. doi:10.1200/JCO.2014.60.7366

120. Borget I, Bonastre J, Catargi B, et al. Quality of life and cost-effectiveness assessment of radioiodine ablation strategies in patients with thyroid cancer: results from the randomized phase III ESTIMABL trial. J Clin Oncol. 2015;33(26):2885-2892. doi:10.1200/JCO.2015.61.6722

121. Hofheinz R-D, Gencer D, Schulz H, et al. Mapisal versus urea cream as prophylaxis for capecitabine-associated hand-foot syndrome: a randomized phase III trial of the AIO Quality of Life Working Group. J Clin Oncol. 2015;33(22):2444-2449. doi:10.1200/JCO.2014.60.4587

122. Brundage M, Sydes MR, Parulekar WR, et al. Impact of radiotherapy when added to androgen-deprivation therapy for locally advanced prostate cancer: long-term quality-of-life outcomes from the NCIC CTG PR3/MRC PR07 randomized trial. J Clin Oncol. 2015;33(19):2151-2157. doi:10.1200/JCO.2014.57.8724

123. Taphoorn MJB, Henriksson R, Bottomley A, et al. Health-related quality of life in a randomized phase III study of bevacizumab, temozolomide, and radiotherapy in newly diagnosed glioblastoma. J Clin Oncol. 2015;33(19):2166-2175. doi:10.1200/JCO.2014.60.3217

124. Taphoorn MJB, Dirven L, Kanner AA, et al. Influence of treatment with tumor-treating fields on health-related quality of life of patients with newly diagnosed glioblastoma: a secondary analysis of a randomized clinical trial. JAMA Oncol. 2018;4(4):495-504. doi:10.1001/jamaoncol.2017.5082

125. Cirkel GA, Hamberg P, Sleijfer S, et al; Dutch WIN-O Consortium. Alternating treatment with pazopanib and everolimus vs continuous pazopanib to delay disease progression in patients with metastatic clear cell renal cell cancer: the ROPETAR randomized clinical trial. JAMA Oncol. 2017;3(4):501-508. doi:10.1001/jamaoncol.2016.5202

126. Melisko ME, Goldman ME, Hwang J, et al. Vaginal testosterone cream vs estradiol vaginal ring for vaginal dryness or decreased libido in women receiving aromatase inhibitors for early-stage breast cancer: a randomized clinical trial. JAMA Oncol. 2017;3(3):313-319. doi:10.1001/jamaoncol.2016.3904

© 2020 Haslam A et al. *JAMA Network Open*.

127. Siu LL, Waldron JN, Chen BE, et al. Effect of standard radiotherapy with cisplatin vs accelerated radiotherapy with panitumumab in locoregionally advanced squamous cell head and neck carcinoma: a randomized clinical trial. JAMA Oncol. 2017;3(2):220-226. doi:10.1001/jamaoncol.2016.4510

128. Awada A, Colomer R, Inoue K, et al. Neratinib plus paclitaxel vs trastuzumab plus paclitaxel in previously untreated metastatic ERBB2-positive breast cancer: the NEfERT-T randomized clinical trial. JAMA Oncol. 2016;2(12):1557-1564. doi:10.1001/jamaoncol.2016.0237

129. Movsas B, Hu C, Sloan J, et al. Quality of life analysis of a radiation dose-escalation study of patients with non-small-cell lung cancer: a secondary analysis of the Radiation Therapy Oncology Group 0617 randomized clinical trial. JAMA Oncol. 2016;2(3):359-367. doi:10.1001/jamaoncol.2015.3969

130. Shaitelman SF, Schlembach PJ, Arzu I, et al. Acute and short-term toxic effects of conventionally fractionated vs hypofractionated whole-breast irradiation: a randomized clinical trial. JAMA Oncol. 2015;1(7):931-941. doi:10.1001/jamaoncol.2015.2666

131. Esplen MJ, Wong J, Warner E, Toner B. Restoring Body Image After Cancer (ReBIC): results of a randomized controlled trial. J Clin Oncol. 2018;36(8):749-756. doi:10.1200/JCO.2017.74.8244

132. Urech C, Grossert A, Alder J, et al. Web-based stress management for newly diagnosed patients with cancer (STREAM): a randomized, wait-list controlled intervention study. J Clin Oncol. 2018;36(8):780-788. doi:10.1200/JCO.2017.74.8491

133. Greer JA, Jacobs JM, El-Jawahri A, et al. Role of patient coping strategies in understanding the effects of early palliative care on quality of life and mood. J Clin Oncol. 2018;36(1):53-60. doi:10.1200/JCO.2017.73.7221

134. El-Jawahri A, Traeger L, Greer JA, et al. Effect of inpatient palliative care during hematopoietic stem-cell transplant on psychological distress 6 months after transplant: results of a randomized clinical trial. J Clin Oncol. 2017;35(32):3714-3721. doi:10.1200/JCO.2017.73.2800

135. van de Wal M, Thewes B, Gielissen M, Speckens A, Prins J. Efficacy of blended cognitive behavior therapy for high fear of recurrence in breast, prostate, and colorectal cancer survivors: the SWORD study, a randomized controlled trial. J Clin Oncol. 2017;35(19):2173-2183. doi:10.1200/JCO.2016.70.5301

136. Maly RC, Liang LJ, Liu Y, Griggs JJ, Ganz PA. Randomized controlled trial of survivorship care plans among low-income, predominantly Latina breast cancer survivors. J Clin Oncol. 2017;35(16):1814-1821. doi:10.1200/JCO.2016.68.9497

137. Hummel SB, van Lankveld JJDM, Oldenburg HSA, et al. Efficacy of internet-based cognitive behavioral therapy in improving sexual functioning of breast cancer survivors: results of a randomized controlled trial. J Clin Oncol. 2017;35(12):1328-1340. doi:10.1200/JCO.2016.69.6021

138. Temel JS, Greer JA, El-Jawahri A, et al. Effects of early integrated palliative care in patients with lung and GI cancer: a randomized clinical trial. J Clin Oncol. 2017;35(8):834-841. doi:10.1200/JCO.2016.70.5046

© 2020 Haslam A et al. *JAMA Network Open*.

139. Chambers SK, Occhipinti S, Foley E, et al. Mindfulness-based cognitive therapy in advanced prostate cancer: a randomized controlled trial. J Clin Oncol. 2017;35(3):291-297. doi:10.1200/JCO.2016.68.8788

140. Bray VJ, Dhillon HM, Bell ML, et al. Evaluation of a web-based cognitive rehabilitation program in cancer

survivors reporting cognitive symptoms after chemotherapy. J Clin Oncol. 2017;35(2):217-225. doi:10.1200/JCO.2016.67.8201

141. Dieng M, Butow PN, Costa DS, et al. Psychoeducational intervention to reduce fear of cancer recurrence in people at high risk of developing another primary melanoma: results of a randomized controlled trial. J Clin Oncol. 2016;34(36):4405-4414. doi:10.1200/JCO.2016.68.2278

142. Johannsen M, O'Connor M, O'Toole MS, Jensen AB, Højris I, Zachariae R. Efficacy of mindfulness-based cognitive therapy on late post-treatment pain in women treated for primary breast cancer: a randomized controlled trial. J Clin Oncol. 2016;34(28):3390-3399. doi:10.1200/JCO.2015.65.0770

143. Lengacher CA, Reich RR, Paterson CL, et al. Examination of broad symptom improvement resulting from mindfulness-based stress reduction in breast cancer survivors: a randomized controlled trial. J Clin Oncol. 2016;34(24):2827-2834. doi:10.1200/JCO.2015.65.7874

144. Kinney AY, Steffen LE, Brumbach BH, et al. Randomized noninferiority trial of telephone delivery of BRCA1/2 genetic counseling compared with in-person counseling: 1-year follow-up. J Clin Oncol. 2016;34(24):2914-2924. doi:10.1200/JCO.2015.65.9557

145. Basch E, Deal AM, Kris MG, et al. Symptom monitoring with patient-reported outcomes during routine cancer treatment: a randomized controlled trial. J Clin Oncol. 2016;34(6):557-565. doi:10.1200/JCO.2015.63.0830

146. Nicolaije KAH, Ezendam NP, Vos MC, et al. Impact of an automatically generated cancer survivorship care plan on patient-reported outcomes in routine clinical practice: longitudinal outcomes of a pragmatic, cluster randomized trial. J Clin Oncol. 2015;33(31):3550-3559. doi:10.1200/JCO.2014.60.3399

147. van den Berg SW, Gielissen MF, Custers JA, van der Graaf WT, Ottevanger PB, Prins JB. BREATH: web-based self-management for psychological adjustment after primary breast cancer—results of a multicenter randomized controlled trial. J Clin Oncol. 2015;33(25):2763-2771. doi:10.1200/JCO.2013.54.9386

148. Epstein RM, Duberstein PR, Fenton JJ, et al. Effect of a patient-centered communication intervention on oncologist-patient communication, quality of life, and health care utilization in advanced cancer: the VOICE randomized clinical trial. JAMA Oncol. 2017;3(1):92-100.

149. Zick SM, Sen A, Wyatt GK, Murphy SL, Arnedt JT, Harris RE. Investigation of 2 types of self-administered acupressure for persistent cancer-related fatigue in breast cancer survivors: a randomized clinical trial. JAMA Oncol. 2016;2(11):1470-1476. doi:10.1001/jamaoncol.2016.1867

© 2020 Haslam A et al. *JAMA Network Open*.

150. Grudzen CR, Richardson LD, Johnson PN, et al. Emergency department-initiated palliative care in advanced cancer: a randomized clinical trial. JAMA Oncol. 2016;2(5):591-598. doi:10.1001/jamaoncol.2015.5252

151. Clemons M, Bouganim N, Smith S, et al. Risk model-guided antiemetic prophylaxis vs physician's choice in patients receiving chemotherapy for early-stage breast cancer: a randomized clinical trial. JAMA Oncol. 2016;2(2):225-231. doi:10.1001/jamaoncol.2015.3730

© 2020 Haslam A et al. *JAMA Network Open*.
